# A novel classification for evaluating episiotomy practices: application to the Burgundy perinatal network

**DOI:** 10.1186/s12884-019-2424-2

**Published:** 2019-08-16

**Authors:** Thomas Desplanches, Emilie Szczepanski, Jonathan Cottenet, Denis Semama, Catherine Quantin, Paul Sagot

**Affiliations:** 1 CHRU Dijon, Department of gynecology, obstetrics, fetal medicine and infertility, Dijon, France; 2 Obstetrical, Perinatal, and Pediatric Epidemiology Team, Epidemiology and Biostatistics Sorbonne Paris Cité Research Center (U1153), INSERM, Paris, France, Paris Descartes University, Paris, France; 3 Service de Biostatistique et d’Informatique Médicale (DIM), Dijon University Hospital, F-21000 Dijon, France; 4 Inserm, CIC 1432, Clinical Epidemiology Unit Dijon, France; Clinical Investigation Center, Clinical Epidemiology Unit, Dijon University Hospital, Dijon, France; 5 CHRU Dijon, Department of Neonatal Pediatrics, Dijon University Hospital, Dijon, France; 6 Biostatistics, Biomathematics, Pharmacoepidemiology and Infectious Diseases (B2PHI), INSERM, UVSQ, Institut Pasteur, Université Paris-Saclay, Paris, France; 7 University of Burgundy, Dijon, France

**Keywords:** Episiotomy, Vaginal delivery, OASIS, Ascending hierarchical classification

## Abstract

**Background:**

Though the rate of episiotomy has decreased in France, the overall episiotomy rate was 20% in the 2016 national perinatal survey. We aimed to develop a classification to facilitate the analysis of episiotomy practices and to evaluate whether episiotomy is associated with a reduction in the rate of obstetric anal sphincter injuries (OASIS) for each subgroup.

**Methods:**

This population-based study included all the deliveries that occurred in the Burgundy Perinatal Network from 2011 to 2016. The main outcome was episiotomy, which was identified thanks to the French Common Classification of Medical Procedures. An ascending hierarchical cluster analysis was performed to build the classification. A clinical audit using the classification was conducted yearly in all obstetric units. The episiotomy rates were described throughout the study period for each subgroup of the classification. The OASIS rates were evaluated by subgroup and the association between mediolateral episiotomy and OASIS was investigated for each subgroup.

**Results:**

Our analyses included 81,290 pregnant women. The classification comprised 7 subgroups: _(1)_ nulliparous single cephalic at term, _(2)_ nulliparous single cephalic at term with instrumental delivery, _(3)_ multiparous single cephalic at term, _(4)_ multiparous single cephalic at term with instrumental delivery, _(5)_ all preterm deliveries (< 37 weeks gestation), _(6)_ all breech deliveries, _(7)_ all multiple deliveries.

Episiotomy rates ranged from 6.2% in Group 3 to 40.9% in Group 2. From 2011 to 2016, every group except breech deliveries experienced a significant decrease in episiotomy rates, ranging from − 28.1 to − 61.0%.

The prevalence of OASIS was the highest in Groups 2 (3.0%) and 4 (2.2%). Overall OASIS rates did not significantly differ with episiotomy use (*P* = 0.25). However, we found that the use of episiotomy was associated with a reduction in OASIS rates in Groups 1 and 2 (odds ratio 0.6 [95% CI 0.4–0.9] and 0.4 [0.3–0.5], respectively). This reduction was only observed in Group 4 with forceps delivery (odds ratio 0.4 [0.1–0.9]).

**Conclusion:**

We developed the first classification for the evaluation of episiotomy practices based on 7 clinically relevant subgroups. This easy-to-use tool can help obstetricians and midwives improve their practices through self-assessment.

**Electronic supplementary material:**

The online version of this article (10.1186/s12884-019-2424-2) contains supplementary material, which is available to authorized users.

## Background

Several studies have reported the side effects of episiotomy [1–4], insufficient prevention of obstetric anal sphincter injuries (OASIS) [5–7] and even an increased risk of OASIS [8]. Thus the current guidelines recommend limiting the use of episiotomy [9–13], and numerous studies have reported a significant decrease in episiotomy rates [14–17], with wide variations across countries and hospitals [15, 18–24]. A recent systematic review reported that routine episiotomy increases the risk of severe perineal trauma and does not reduce the risk of OASIS. However, the results are still uncertain in case of instrumental vaginal delivery [7].

In most studies, episiotomy rates were compared either in a whole population of pregnant women or in subgroups based on parity and/or mode of delivery.

We hypothesized that a more detailed classification based on well-defined subgroups of women and obstetrical practices, similar to that used to compare cesarean practices [25], could allow a better assessment in the context of restrictive episiotomy policies, and could be used to decrease the episiotomy rate.

Using the Burgundy Perinatal Network (BPN) database, the main objective of this study was to build a classification in order to analyze episiotomy practices accurately. The second objective was to evaluate whether episiotomy is associated with a reduction in OASIS rates according to the subgroups of the classification.

## Methods

### Data source

Since 2000, all deliveries and terminations of pregnancies that occur within the BPN at or after 22 completed weeks of gestation and/or with a birthweight > 500 g have been systematically recorded in an anonymous database used to assess medical practices within the network [26] (Authorization- Commission Nationale Informatique et Liberté - n° 455,451). The BPN database covers all public and private hospitals in Burgundy, a French region with approximately 1,600,000 inhabitants. Maternal and neonatal medical data are prospectively recorded from the mandatory discharge abstracts for each hospitalized woman (used to determine the activity-based funding of hospitals in France). Twenty additional specific perinatal indicators, 11 per mother and 9 per newborn, were also prospectively recorded. Data entry was overseen by the physicians in the medical records department, and our statistician compared the records compiled in our database to the birthing room registry in order to ensure exhaustiveness. Statistical coherence was evaluated, and any discrepancies were reported to the medical team and amended. In accordance with European and French law, patient data have to be rendered anonymous. The anonymization methods routinely used for BPN data were developed by our research team (ANONYMAT Software also used in national applications) [27, 28].

### Study design and population

A retrospective observational study was conducted in the 13 maternity wards of the hierarchical BPN between January 2011 and December 2016. Levels of care are based on a three-tiered system which includes level-3 hospitals (defined as the highest level of care for preterm birth), other neonatal units, called level-2 units, which are equipped to manage preterm birth from 32 to 36 completed weeks of gestation, and maternity units without a neonatal unit (level-1 units) [29]. Over this 6-year period, the 13 BPN maternity wards managed approximately 17,000 births per year, distributed as follows: 3000 births in level-1 maternity wards, 11,000 births in level-2 maternity wards and 3000 births in the single level-3 maternity ward (university hospital).

In the current study, we excluded deliveries at less than 25 weeks of gestation, termination of pregnancy (defined as abortion for a maternal and/or therapeutic fetal indication), home birth, and caesarean deliveries.

### Variables and definitions

The following maternal and obstetrical characteristics were considered: parity (nulliparous or multiparous women), type of pregnancy (single or multiple pregnancies), fetal presentation at birth (cephalic, breech or other), and the mode of delivery defined as vaginal, instrumental (forceps/ spatula or vacuum delivery) or caesarean delivery.

In France, obstetricians are supposed to perform all deliveries in private maternity hospitals. However, in some private hospital with public-service tasks and in public maternity hospitals, midwives perform vaginal deliveries in full term cephalic presentation and obstetricians are called in for instrumental delivery, breech, preterm term and caesarean deliveries.

### Outcomes

The primary outcome, mediolateral episiotomy, was identified with the code JMPA006 according to the French Common Classification of Medical Procedures.

The secondary outcome was OASIS. To classify OASIS, we used the Royal College of Obstetricians and Gynecologists classification [12], which is most widely used in the international literature. Only third-degree (defined as injuries of external and/ or internal anal sphincter) and fourth-degree tears (defined as injuries of anal sphincter complex and anorectal mucosa) were taken into account and pooled for the analyses [13]. OASIS was diagnosed by a midwife or obstetrician with a clinical examination (vaginal and rectal examination) of the perineum just after delivery, and the repair was performed by an obstetrician. OASIS was identified in our database using the International Classification of Diseases 10th Revision (O702 and O703) or the French national hospital discharge database (JMCA001 and JMCA003).

### Statistical analysis

Univariate analyses were performed to describe the population characteristics, including changes in the instrumental delivery rate and the cesarean delivery rate, using the Cochran-Armitage test.

To build the classification, we used the principle of a classification system [25]. These systems are used in medicine to transform crude data into information that can be used to improve care, as Robson et al. did for caesarean sections [30]. Our classification was built from data recorded in 2011 and 2012. First, a multiple correspondence analysis was performed using clinically relevant variables or variables known to be associated with episiotomy [18, 23, 31–34]: type of pregnancy, parity, fetal presentation, gestational age, and mode of delivery. A final ascending hierarchical classification using Ward’s step method was then performed for the first 4 dimensions resulting from multiple correspondence analysis (65.4% of the inertia) to establish the classification. The ascending hierarchical classification, conventionally used in other medical specialties [35], maximizes homogeneity among the clusters produced by classification, and, at the same time, maximizes the heterogeneity between them. The number of clusters was chosen using the curve of the semi-partial R^2^.

The episiotomy rates, the relative size of each group (i.e. number of women in the group/total number of women delivered) and, finally, each group’s contribution to the episiotomy rate (i.e. number of episiotomies in the group/total number of women having episiotomy) were described by subgroup. An annual audit cycle using the classification was conducted in all maternity wards from 2013. Every year since, the results of the previous year have been presented by the BPN evaluation unit to the medical teams in each maternity ward. A detailed report provides a comparative analysis of the episiotomy rates per obstetric unit. The aim of the report is to identify the differences in practices among the obstetric units in the BPN in order to make appropriate changes in patient management. The consequences of these changes are then assessed the following year.

We used the classification to describe the changes in episiotomy practices from 2011 to 2016, and trends in episiotomy were analyzed using the Cochran-Armitage test. The episiotomy rates were also compared according to the level of the maternity ward [1–3], or [29, 36] and the status (private or public) of the unit with Chi-squared tests.

The OASIS rates, the relative size of each group (i.e. number of women in the group/total number of women delivered) and, finally, each group’s contribution to the OASIS rate (i.e. number of OASIS in the group/total number of women having OASIS) were described by subgroup.

Fisher’s exact tests were used to compare OASIS rates by subgroup, with and without episiotomy.

To investigate the potential effect of episiotomy on OASIS, odds ratios were calculated by subgroup of classification.

Statistical significance was set with a two-tailed test at *p* <  0.05. All analyses were done with SAS v9.4 software.

## Results

A total of 98,053 pregnant women delivered from 2011 to 2016, not including deliveries at less than 25 weeks of gestation, termination of pregnancy and home birth. Since women giving birth by caesarean section (*n* = 16,763) were also excluded, 81,290 pregnant women were finally included in our analyses. The mean annual cesarean delivery rate was 17% and did not differ significantly during the study period (Cochran-Armitage test *P* = 0.14). Patient demographic data is presented per year (Additional file 1: Table S1).

### Proposal of a classification for episiotomy practices

Using data from the BPN from 2011 and 2012, 7 clusters were identified with ascending hierarchical classification. Only 3 of the 5888 women who gave birth by caesarean section had an episiotomy, which led us to exclude these women.

Nulliparous women with a single cephalic pregnancy at term with non-instrumental delivery and multiparous women with a single cephalic pregnancy at term delivered by instrumental delivery were gathered into one cluster in ascending hierarchical classification. For increased clinical relevance, we decided to split the cluster into two groups. The final classification includes 7 groups (Table 1).

**Table 1:** Classification of episiotomy practices: Burgundy perinatal network data, vaginal deliveries, 2011–2012

Classification of episiotomy practices	Number of episiotomies / Number of women	Episiotomy rate of each group (%)	Relative size of each group (%)^a^	Contribution of each group to episiotomy rate (%) ^b^
1 – Nulliparous women with a single cephalic pregnancy at ≥37 weeks gestation, non-instrumental delivery	1942/ 7987	24.3	28.7	39.3
2 – Nulliparous women with a single cephalic pregnancy at ≥37 weeks gestation, instrumental delivery	1381/ 2972	46.5	10.7	28.0
2a – Forceps/ spatula delivery	801/ 1223	65.5	4.4	16.2
2b – Vacuum delivery	580/ 1749	33.2	6.3	11.8
3 – Multiparous women with a single cephalic pregnancy at ≥37 weeks gestation, non- instrumental delivery	1066/ 14,237	7.5	51.2	21.6
4 – Multiparous women with a single cephalic pregnancy at ≥37 weeks gestation, instrumental delivery	250/ 771	32.4	2.8	5.1
4a – Forceps/ spatula delivery	140/ 247	56.7	0.9	2.8
4b – Vacuum delivery	110/ 524	21.0	1.9	2.2
5 – All women with a single cephalic pregnancy at < 37 weeks gestation	164/ 1172	14.0	4.2	3.3
6 – All women with a single breech pregnancy	72/ 374	19.3	1.3	1.5
7 – All women with multiple pregnancy	61/ 270	22.6	1.0	1.2
Total	4936/ 27,783	17.8	100	100

^a^number of women in the group / total number of women delivered

^b^number of episiotomies in the group / total number of women having episiotomy

Approximately 94% of episiotomies were performed on women with a single cephalic pregnancy at or above 37 weeks of gestation (Groups 1 to 4). Rates of episiotomy were substantially affected by the use of instrumental delivery and by parity (7.5% in multiparous women without instrumental delivery vs. 46.5% in nulliparous women with instrumental delivery, Chi-squared test *p* <  0.001).

For instrumental delivery (Groups 2 and 4), episiotomy rates varied with the type of instrument (forceps or vacuum delivery, Chi-squared tests p <  0.001) and were particularly high with the use of forceps in nulliparous women (65.5%).

### Use of the classification in a perinatal network: description of the changes in episiotomy rates from 2011 to 2016

The overall rate of episiotomy was 15.5%, (Table 2) but it varied widely among maternity wards with figures ranging from 7.5 to 35.7% (Additional file 2: Table S2).

**Table 2:** Change in episiotomy rates according to classification: Burgundy perinatal network data, vaginal deliveries, 2011–2016

Classification of episiotomy practices	Episiotomy rates (%)									
	Pooled 2011–2016	2011	2012	2013	2014	2015	2016	*P* ^*a*^	Absolute Difference 2011–2016	Relative Difference (%) 2011–2016
1 – Nulliparous women with a single cephalic pregnancy, at ≥37 weeks gestation, non-instrumental delivery	21.8	22.8	26.0	24.2	22.0	18.1	16.4	< 0.001	−6.4	−28.1
2 – Nulliparous women with a single cephalic pregnancy at ≥37 weeks gestation, instrumental delivery	40.9	46.8	46.1	45.4	40.7	34.7	33.0	< 0.001	−13.8	−29.5
2a – Forceps/ spatula delivery	56.0	66.5	64.5	64.2	57.9	45.1	41.6	< 0.001	−24.9	−37.4
2b – Vacuum delivery	30.2	33.9	32.2	32.5	29.6	27.4	25.8	< 0.001	−8.1	−23.9
3 – Multiparous women with a single cephalic pregnancy at ≥37 weeks gestation, non- instrumental delivery	6.2	7.8	7.2	6.4	5.9	5.1	4.5	< 0.001	−3.3	−42.3
4 – Multiparous women with a single cephalic pregnancy at ≥37 weeks gestation, instrumental delivery	26.8	33.5	31.4	32.1	23.6	21.3	21.5	< 0.001	−12.0	−35.8
2a – Forceps/ spatula delivery	46.2	61.2	53.5	55.6	41.7	34.7	37.2	< 0.001	−24.0	−39.2
2b – Vacuum delivery	17.8	23.1	18.7	22.1	16.0	14.9	13.6	< 0.001	−9.5	−41.2
5 – All women with a single cephalic pregnancy at < 37 weeks gestation	11.1	14.7	13.3	13.5	8.7	9.0	6.9	< 0.001	−7.8	− 53.1
6 – All women with a single breech pregnancy	17.7	21.8	16.6	15.1	21.4	15.7	16.4	0.24	−5.4	−24.8
7 – All women with multiple pregnancy	17.5	25.4	19.9	20.8	18.2	9.9	9.9	0.001	−15.5	−61.0
Total	15.5	17.8	17.8	16.7	15.0	12.9	12.1	< 0.001	−5.7	−32.0

^a^Cochran Armitage Test

Despite a 3.2% increase in the rate of instrumental delivery between 2011 and 2016 (Additional file 1: Table S1), we observed a 32.0% decrease in the overall rate of episiotomy (Table 2). The reduction was more pronounced for multiple pregnancies (Group 7; − 61.0%) and for preterm birth (Group 5; − 53.1%). For each group, except for breech birth (Group 6), the Cochran-Armitage test revealed a significant decrease in episiotomy rates (Table 2). In addition, in level-2 and level-3 maternity wards, a significant decrease in episiotomy rates was shown in all but Group 6 (breech birth) between 2011 and 2016, whereas level 1 maternity wards only saw a decrease for Groups 1, 3 and 5 (Additional file 3: Table S3).

### Variations in episiotomy practices according to place of birth and hospital status

The level of the maternity ward also had a significant effect on episiotomy rates with an overall incidence of 12.0% in level-3, 15.6% in level-2 and 17.9% in level-1 maternities. The single level-3 maternity reported the lowest rates of episiotomy in all groups except multiple pregnancies (Additional file 4: Table S4).

The episiotomy rates for instrumental delivery (Groups 2 and 4) were significantly higher in level-1 maternity wards than in level-2 and 3 maternity wards and did not decrease over time (Additional file 4: Table S4).

Hospital status slightly affected the rates of episiotomy in some groups of patients. The episiotomy rate in private hospitals was significantly lower in Group 1 but higher in Group 2 (Additional file 5: Table S5).

### OASIS according to the classification for episiotomy practices

The overall rate of OASIS remains low (0.9%) but increased significantly during the study period, from 0.8 to 1.1% (Additional file 1: Table S1). The prevalence of OASIS was higher in Group 1 (1.1%), 2 (3.0%) and 4 (2.2%), and lower (less than 0.5%) in Groups 3, 5, 6 and 7. More than 70% of OASIS occurred in Groups 1 and 2 (Table 3).

**Table 3:** OASIS according to classification of episiotomy practices, vaginal deliveries, 2011–2016

Classification of episiotomy practices	Number of OASIS / Number of women	OASIS rate of each group (%)	Relative size of each group (%)^a^	Contribution of each group to OASIS rate (%) ^b^
1 – Nulliparous women with a single cephalic pregnancy, at ≥37 weeks gestation, non-instrumental delivery	243/ 22,239	1.1	27.4	33.9
2 – Nulliparous women with a single cephalic pregnancy at ≥37 weeks gestation, instrumental delivery	271/ 8984	3.0	11.1	37.8
3 – Multiparous women with a single cephalic pregnancy at ≥37 weeks gestation, non- instrumental delivery	122/ 41,929	0.3	51.6	17.0
4 – Multiparous women with a single cephalic pregnancy at ≥37 weeks gestation, instrumental delivery	60/ 2677	2.2	3.3	8.4
5 – All women with a single cephalic pregnancy at < 37 weeks gestation	12/ 3374	0.4	4.2	1.7
6 – All women with a single breech pregnancy	5/ 1268	0.4	1.6	0.7
7 – All women with multiple pregnancy	4/ 819	0.5	1.0	0.6
Total	717/ 81,290	0.9	100	100

*OASIS* obstetric anal sphincter injuries

^a^number of women in the group / total number of women delivered

^b^number of OASIS in the group / total number of women having OASIS

The overall OASIS rate was not associated with episiotomy status (*P* = 0.25). OASIS was lower in Groups 1, 2 and 4a with episiotomy than in Groups 1, 2 and 4a without episiotomy (odds ratio 0.6, 95% CI [0.4–0.9], 0.4 [0.3–0.5] and 0.4 [0.1–0.9], respectively) (Table 4).

**Table 4:** Comparison of OASIS with or without episiotomy according to classification, vaginal deliveries, 2011–2016

Classification of episiotomy practices	OASIS						
	Without episiotomy		With episiotomy		*P* ^*a*^	OR IC 95%	*P*
	n/N	%	n/N	%			
1 – Nulliparous women with a single cephalic pregnancy, at ≥37 weeks gestation, non- instrumental delivery	206/17,395	1.2	37/4844	0.8	0.01	0.6 [0.4–0.9]	0.01
2 – Nulliparous women with a single cephalic pregnancy, at ≥37 weeks gestation, instrumental delivery	212/5307	4.0	59/3677	1.6	0.001	0.4 [0.3–0.5]	0.001
2a -forceps/ spatula delivery	101/1642	6.2	46/2089	2.2	0.001	0.3 [0.2–0.5]	0.001
2b -vacuum delivery	111/3665	3.0	13/1588	0.8	0.001	0.3 [0.1–0.5]	0.001
3 - Multiparous women with a single cephalic pregnancy, at ≥37 weeks gestation, non-instrumental delivery	110/39,347	0.3	12/2582	0.5	0.09	1.7 [0.9–3.0]	0.09
4 – Multiparous women with a single cephalic pregnancy, at ≥37 weeks gestation, instrumental delivery	51/1960	2.6	9/717	1.3	0.04	0.5 [0.2–0.9]	0.04
4a - forceps/ spatula delivery	18/455	4.0	6/390	1.5	0.04	0.4 [0.1–0.9]	0.04
4b - vacuum delivery	33/1505	2.2	3/327	0.9	0.18	0.4 [0.1–1.3]	0.14
5 – All women with a single cephalic pregnancy, at < 37 weeks gestation	10/3000	0.3	2/374	0.5	0.63	1.6 [0.3–7.4]	0.54
6 – All women with a single breech pregnancy	4/1043	0.4	1/225	0.4	1	1.2 [0.1–10.4]	0.89
7 – All women with multiple pregnancy	2/676	0.3	2/143	1.4	0.14	4.8 [0.7–34.2]	0.11
Total	595/68,728	0.9	122/12,562	1.0	0.25	1.1 [0.9–1.4]	0.24

*OASIS* obstetric anal sphincter injuries. *OR* odds ratio

^a^Fisher’s exact tests

## Discussion

In this study, we propose the first classification for episiotomy practices. It consists of 7 well-defined, prospective, mutually exclusive and clinically relevant subgroups, allowing a more accurate assessment of episiotomy practices, whatever the level of specialization of the maternity ward or the type of healthcare professional. We showed more precisely how episiotomy rates varied across subgroups. OASIS was more prevalent in Group 1 (1.1%), 2 (3.0%) and Group 4 (2.2%). We found that OASIS was significantly lower in Groups 1, 2 and 4a when an episiotomy was performed.

Previous studies [14, 19–22] have limited their analyses to overall episiotomy rates or in case of instrumental delivery [37, 38], but no classification has been used so far. The first 4 groups of our classification combine four parameters: parity, term, presentation and mode of delivery. Most of these characteristics were already used by Robson et al. as parameters to determine the classification of caesarean sections [25], but Robson’s classification does not take into account the mode of vaginal delivery (with or without instrumental delivery), which is a major parameter for accurately analyzing episiotomy practices.

The combination of such characteristics is of major importance to accurately analyze practices because the episiotomy rates varied broadly: multiparous women at term with cephalic presentation and without instrumental delivery (Group 3) had the lowest rate of episiotomy, while nulliparous women at term with cephalic presentation and with instrumental delivery (Group 2) had the highest rate. Even if the other groups [5–7], and accounted for only 6% of all episiotomies, our results highlight a significant rate in instances of prematurity, breech birth and multiple pregnancy, which are frequently omitted from randomized trials or cohort studies [7, 14, 18, 22, 39, 40].

Our classification also allowed us to further analyze the rate of episiotomy depending on the type of instrumental delivery used (forceps or vacuum delivery in Groups 2 and 4). Our results clearly show that forceps are associated with a higher rate of episiotomy. This partially explains the high episiotomy rate in level-1 Burgundy maternity wards, where forceps are mainly used.

The use of our classification also makes it possible to distinguish the episiotomy practices of obstetricians and midwives. For example, Groups 1 and 3 can be used to evaluate the episiotomy practices of midwives in public hospitals, while Groups 2 and 4 reflect the practices of obstetricians in public and private hospitals.

Previous research [18] has shown a variation in episiotomy by place of birth using an overall episiotomy rate. Our classification provides a more detailed analysis by specifying precisely in which subgroups the differences were observed.

As reported previously [15, 17, 23, 24, 41], we observed a significant decrease in the overall episiotomy rate, but, in addition, we were able to accurately describe changes over time in each subgroup. For example, although there is no evidence indicating that an episiotomy in breech presentation prevents OASIS [9], the episiotomy rate for breech deliveries remained stable during the study period. We also did not observe a reduction of episiotomy practices among obstetricians practicing in level-1 Burgundy maternity wards. These example suggest that the implementation of evidence-based practices remains a significant challenge which requires comprehensive approaches at different levels [42]. As shown by Althabe et al. [43], reducing a common practice such as episiotomy is difficult.

The decrease in episiotomy rates can be explained in part by the application of national French obstetrical guidelines [44], but, above all, these recommendations were actively disseminated within the BPN through our annual audit cycle. Our hypothesis was that the use of a classification that takes into accounts both maternal characteristics and obstetrical practices would facilitate the comparison of episiotomy practices and allow healthcare professionals to self-assess their episiotomy practices. Other factors may have contributed to lower episiotomy rates, and the absence of a control group does not allow us to establish a causal link. Interestingly, the 2016 French national perinatal survey found that our region has the lowest episiotomy rate in France (the national average is roughly 20%) [45].

Similar to previous studies [41, 46, 47], an increase in OASIS was observed. This rise could be associated with the decrease in episiotomy rates [41], but may also be influenced by the increases in instrumental delivery and nulliparity which are known risk factors for OASIS [8]. Approximately 70% of OASIS occurred in nulliparous women with a single cephalic pregnancy at term, without instrumental delivery (Group 1) or with instrumental delivery (Group 2). Our results confirm well-known risk factors for OASIS, i.e. nulliparity and instrumental delivery ^44^. We also found a lower rate of OASIS when episiotomy use was more frequent, suggesting that episiotomy has a protective effect in these subgroups, in particular for nulliparous women with a single cephalic pregnancy at term, with forceps delivery (Group 2a).

Only one randomized controlled trial comparing restrictive versus routine use of mediolateral episiotomy has been published [48]: the authors found no effect on OASIS, but the sample size was inadequate according to recent American guidelines [13].

The protective effect of mediolateral episiotomy is still debated in case in of instrumental delivery [7, 32, 38]. Several international guidelines highlight that mediolateral episiotomy should be considered in instrumental deliveries [9, 12]. This protective effect is evident in our results (with a low prevalence of episiotomy) as well as in a recent study by Van Bavel et al. [37] which reported a high prevalence of episiotomy. We found that episiotomy can have a protective effect in both forceps and vacuum delivery in nulliparous women (Groups 1 and 2), but only for forceps/ spatula delivery in multiparous women. Our results are similar to the results of Raïsanen et al., who investigated the effects of lateral episiotomy in women delivered with vacuum in Finland [49] but diverge from those of Van Bavel et al. [37]. In order to limit a potential indication bias, we stratified using a classification, but only the main confounding factors have been taken into account. The information about the methods of preventing perineal injury were not recorded in our database, but the manual control of the expulsion of the fetus at the end of the second stage of labor is almost systematic in France [50]. Further studies are required to assess the protective effects of episiotomy in subgroups at high risk of OASIS.

By contrast, the episiotomy rate can be safely reduced in groups 3, 5, 6, and 7 in which the very low prevalence of OASIS is not influenced by this practice. These groups represent approximately 60% of the population for whom a restrictive episiotomy policy should always be encouraged. Studies conducted by Räisänen et al. [41] and by Rosen et al. [31] have shown that it is possible to reduce the episiotomy rate in low-risk women.

The main strength of our study is that our classification was constructed from a large cohort of pregnant women giving birth in different levels [1–3], and and types (public and private) of maternity wards, thus encouraging its generalized use. Our classification respects the principles of classification systems and was based on clearly defined characteristics that are systematically collected for medical records, facilitating the assessment of episiotomy rates across different settings. The parameters (parity, term, presentation and mode of delivery for example) used are carefully defined, accurately and systematically collected. All pregnant women were assigned to a single group and could only belong to one group at a time.

The main potential limitation of this study is the identification of episiotomy and OASIS through a national hospital discharge database. However, a 2012 validation study carried out in 3 university hospitals to evaluate the metrological quality of hospital discharge abstracts for perinatal indicators reported a positive predictive value of 88.9% [79.7–98.1] and a sensitivity of 90.9% [82.4–99.4] for episiotomy, whatever the mode of delivery [51]. In addition, the overall prevalence of OASIS in our study was comparable to the prevalence reported in the 2016 French national perinatal survey (0.9 versus 0.8, respectively).

The French national guidelines have recommended that median episiotomy no longer be performed since 2005 [44]. Though the type of episiotomy is not recorded, all the obstetricians and midwives in the BPN used only mediolateral episiotomies, as recommended. This information is consistent with the most recently published French national perinatal survey results which confirmed that the use of median episiotomy is rare [52].

Another limitation is that our classification did not take into account vaginal birth after cesarean section or the occiput posterior position, but given the very low prevalence of these two parameters, they were not included in our classification.

This classification does not address the indication of episiotomy which could also be of interest. However, classifications based on indications for episiotomy have some limits: poor definitions, groups which are not mutually exclusive and a need to rank the indications. A systematic review comparing the different type of classifications used for caesarean section [53] showed that classifications based on women’s characteristics were the most appropriate. In addition, studies dealing with episiotomy indications disclosed that these indications are subjective, not consistent with international practice guidelines [12, 13, 54], variable by country [14, 22], and dependent on the type of obstetrical staff involved [55]. They also reported that many of the indications reported by healthcare professionals are not congruent with international clinical guidelines [54].

## Conclusion

We have developed a classification of episiotomy practices based on 7 clinically relevant subgroups, allowing an accurate analysis of episiotomy practices. The use of this classification has provided a better understanding of the episiotomy practices for each type of maternity hospital. Our analysis revealed that changes in episiotomy practices varied by subgroup, and we were able to define groups with a low risk of OASIS in whom the use of episiotomy can be safely reduced.

Using this classification would constitute a solid first step in the quality process for improving the practices of episiotomy in all clinical circumstances. This classification can be easily implemented at regional or national levels, because its use is simple, reproducible and does not require sophisticated software.

## Additional files

Additional file 1: **Table S1.** Change in maternal, obstetrical and neonatal characteristics for pregnant women giving birth, 2011–2016. (DOCX 21 kb)

Additional file 2: **Table S2.** Episiotomy rates by maternity wards (%): Burgundy perinatal network data, vaginal deliveries, 2011–2016. (DOCX 21 kb)

Additional file 3: **Table S3.** Change in episiotomy rates with time by level of maternity wards (%): Burgundy perinatal network data, vaginal deliveries, 2011–2016. (DOCX 22 kb)

Additional file 4: **Table S4.** Comparison of episiotomy rates by level of maternity ward according to classification: Burgundy perinatal network data, vaginal deliveries, 2011–2016. (DOCX 19 kb)

Additional file 5: **Table S5.** Comparison of episiotomy rates according to hospital status: Burgundy perinatal network data, vaginal deliveries, 2011–2016. (DOCX 18 kb)

## Data Availability

The datasets used and/or analyzed during the current study are available from the corresponding author on reasonable request.

## Abbreviations

BPN: Burgundy Perinatal Network
OASIS: Obstetric anal sphincter injuries
