# Supplementary material for: A novel classification for evaluating episiotomy practices: application to the Burgundy perinatal network
Source: BMC Pregnancy Childbirth. 2019 Aug 16;19:300. doi: 10.1186/s12884-019-2424-2 (PMC6698013; doi:10.1186/s12884-019-2424-2)
Supplement: Supplementary file 1 — Table S1. Change in maternal, obstetrical and neonatal characteristics for pregnant women giving birth, 2011–2016. (DOCX 21 kb) [file 12884_2019_2424_MOESM1_ESM.docx]

Additional file 1: Table S1: Change in maternal, obstetrical and neonatal characteristics for pregnant women giving birth, 2011- 2016.

|  |  |  | Years | | | | | | | | | | | | *P** |
| --- | --- | --- | --- | --- | --- | --- | --- | --- | --- | --- | --- | --- | --- | --- | --- |
|  | Pooled  2011-2016 | | 2011 | | 2012 | | 2013 | | 2014 | | 2015 | | 2016 | |  |
|  | n | % | n | % | n | % | n | % | n | % | n | % | n | % |  |
| Parity |  |  |  |  |  |  |  |  |  |  |  |  |  |  |  |
| Nulliparous | 33,833 | 41.6 | 6,121 | 43.3 | 5,694 | 41.8 | 5,655 | 41.0 | 5,575 | 40.8 | 5,476 | 41.1 | 5,312 | 41.8 | 0.005 |
| Multiparous | 47,451 | 58.4 | 8,026 | 56.7 | 7,941 | 58.2 | 8,145 | 59.0 | 8,100 | 59.2 | 7,838 | 58.9 | 7,401 | 58.2 |  |
| Multiple Pregnancy | 819 | 1.0 | 134 | 1.0 | 136 | 1.0 | 144 | 1.0 | 143 | 1.1 | 131 | 1.0 | 131 | 1.0 | 0.59 |
| Fetal Presentation |  |  |  |  |  |  |  |  |  |  |  |  |  |  |  |
| Cephalic | 79,852 | 98.2 | 13,931 | 98.5 | 13,424 | 98.4 | 13,556 | 98.2 | 13,430 | 98.2 | 13,046 | 98.0 | 12,465 | 98.1 | 0.0003 |
| Breech | 1,438 | 1.8 | 218 | 1.5 | 214 | 1.6 | 244 | 1.8 | 246 | 1.8 | 268 | 2.0 | 248 | 1.9 |  |
| Instrumental delivery |  |  |  |  |  |  |  |  |  |  |  |  |  |  |  |
| Forceps | 4,986 | 6.1 | 779 | 5.5 | 822 | 6.0 | 779 | 5.6 | 776 | 5.7 | 859 | 6.4 | 971 | 7.6 | < 0.001 |
| Vacuum | 7,338 | 9.0 | 1,233 | 8.7 | 1,126 | 8.3 | 1,198 | 8.7 | 1,260 | 9.2 | 1,269 | 9.5 | 1,252 | 9.8 | < 0.001 |
| OASIS | 717 | 0.9 | 110 | 0.8 | 107 | 0.8 | 125 | 0.9 | 101 | 0.7 | 139 | 1.0 | 135 | 1.1 | 0.003 |
| Gestational age (Weeks) |  |  |  |  |  |  |  |  |  |  |  |  |  |  |  |
| < 37 | 3,984 | 4.9 | 688 | 4.9 | 681 | 5.0 | 650 | 4.7 | 690 | 5.1 | 627 | 4.7 | 648 | 5.1 | 0.69 |
| ≥ 37 | 77,306 | 95.1 | 13,461 | 95.1 | 12,957 | 95.0 | 13,150 | 95.3 | 12,986 | 94.9 | 12,687 | 95.3 | 12,065 | 94.9 |  |

OASIS: obstetric anal sphincter injuries. *Cochran Armitage Test (2011-2016)
