# Supplementary material for: A novel classification for evaluating episiotomy practices: application to the Burgundy perinatal network
Source: BMC Pregnancy Childbirth. 2019 Aug 16;19:300. doi: 10.1186/s12884-019-2424-2 (PMC6698013; doi:10.1186/s12884-019-2424-2)
Supplement: Supplementary file 2 — Table S2. Episiotomy rates by maternity wards (%): Burgundy perinatal network data, vaginal deliveries, 2011–2016. (DOCX 21 kb) [file 12884_2019_2424_MOESM2_ESM.docx]

Additional file 2: Table S2: Episiotomy rates by maternity wards (%): Burgundy perinatal network data, vaginal deliveries, 2011-2016.

|  | Hospitals | | | | | | | | | | | | |
| --- | --- | --- | --- | --- | --- | --- | --- | --- | --- | --- | --- | --- | --- |
| Groups | A* | B | C | D | E | F | G | H | I | J | K | L* | M |
| 1 | 18.5 | 38.4 | 22.9 | 31.2 | 13.2 | 22.7 | 29.4 | 8.7 | 20.2 | 20.5 | 50.9 | 13.2 | 15.1 |
| 2 | 55.8 | 82.4 | 66.2 | 76.6 | 42.5 | 67.4 | 71.3 | 26.3 | 36.8 | 5.5 | 92.1 | 42.5 | 32.4 |
| 2a | 57.5 | 82.4 | 67.1 | 77.7 | 62.0 | 73.9 | 76.9 | 32.0 | 42.3 | 7.0 | 92.1 | 67.1 | 47.2 |
| 2b | 55.1 | 0.0 | 59.6 | 67.7 | 34.1 | 34.8 | 62.4 | 21.2 | 35.4 | 4.5 | 0.0 | 38.1 | 24.6 |
| 3 | 7.0 | 10.3 | 4.5 | 11.2 | 2.9 | 5.7 | 7.7 | 2.7 | 5.1 | 4.4 | 18.2 | 6.0 | 4.1 |
| 4 | 43.1 | 64.1 | 49.5 | 64.8 | 23.5 | 54.5 | 50.0 | 14.2 | 20.6 | 6.0 | 82.1 | 26.6 | 20.2 |
| 4a | 40.0 | 64.1 | 56.0 | 70.1 | 50.0 | 70.0 | 65.8 | 26.1 | 21.1 | 10.9 | 81.8 | 56.1 | 36.1 |
| 4b | 44.4 | 0.0 | 22.2 | 27.3 | 17.5 | 30.8 | 30.6 | 7.9 | 20.5 | 3.6 | 100.0 | 22.8 | 14.9 |
| 5 | 10.5 | 20.9 | 10.4 | 17.2 | 6.7 | 8.6 | 16.8 | 5.2 | 9.2 | 7.6 | 27.4 | 14.2 | 7.8 |
| 6 | 25.0 | 33.3 | 3.6 | 27.8 | 20.0 | 28.6 | 35.1 | 8.7 | 22.8 | 12.8 | 35.5 | 16.2 | 6.4 |
| 7 | 0.0 | 14.3 | 9.1 | 33.3 | 16.7 | 100.0 | 22.0 | 10.0 | 13.1 | 4.3 | 39.1 | 7.3 | 22.8 |
| Total | 16.7 | 27.2 | 17.1 | 24.9 | 11.3 | 15.7 | 20.2 | 7.5 | 13.4 | 9.2 | 35.7 | 16.0 | 12.0 |

*Private hospitals
