# Supplementary material for: A novel classification for evaluating episiotomy practices: application to the Burgundy perinatal network
Source: BMC Pregnancy Childbirth. 2019 Aug 16;19:300. doi: 10.1186/s12884-019-2424-2 (PMC6698013; doi:10.1186/s12884-019-2424-2)
Supplement: Supplementary file 3 — Table S3. Change in episiotomy rates with time by level of maternity wards (%): Burgundy perinatal network data, vaginal deliveries, 2011–2016. (DOCX 22 kb) [file 12884_2019_2424_MOESM3_ESM.docx]

Additional file 3: Table S3: Change in episiotomy rates with time by level of maternity wards (%): Burgundy perinatal network data, vaginal deliveries, 2011-2016.

| Maternity Wards | 2011 | 2012 | 2013 | 2014 | 2015 | 2016 | *P** |
| --- | --- | --- | --- | --- | --- | --- | --- |
| Level 1 |  |  |  |  |  |  |  |
| 1- Nulliparous women with a single cephalic pregnancy, at ≥ 37 weeks gestation, non-instrumental delivery | 23.9 | 26.1 | 25.1 | 20.5 | 22.5 | 19.0 | 0.003 |
| 2- Nulliparous women with a single cephalic pregnancy at ≥ 37 weeks gestation, instrumental delivery | 57.7 | 60.1 | 65.5 | 61.8 | 71.0 | 61.9 | 0.05 |
| 3-Multiparous women with a single cephalic pregnancy at ≥ 37 weeks gestation, non-instrumental delivery | 8.4 | 8.0 | 6.2 | 5.4 | 5.5 | 4.4 | < 0.0001 |
| 4-Multiparous women with a single cephalic pregnancy at ≥ 37 weeks gestation, instrumental delivery | 54.0 | 46.3 | 55.1 | 37.7 | 38.3 | 50.0 | 0.20 |
| 5-All women with a single cephalic pregnancy at < 37 weeks gestation | 17.6 | 11.0 | 10.5 | 9.1 | 11.4 | 6.3 | 0.03 |
| 6-All women with a single breech pregnancy | 19.0 | 16.7 | 9.7 | 29.4 | 23.1 | 26.9 | 0.24 |
| 7-All women with multiple pregnancy | 19.0 | 26.3 | 9.1 | 20.0 | 16.7 | 30.0 | 0.84 |
| Total | 19.4 | 19.5 | 18.2 | 16.6 | 17.2 | 16.2 | < 0.0001 |
| Level 2 |  |  |  |  |  |  |  |
| 1- Nulliparous women with a single cephalic pregnancy, at ≥ 37 weeks gestation, non-instrumental delivery | 23.2 | 27.0 | 25.6 | 25.3 | 19.5 | 18.5 | < 0.0001 |
| 2- Nulliparous women with a single cephalic pregnancy at ≥ 37 weeks gestation, instrumental delivery | 43.6 | 40.7 | 41.0 | 36.2 | 32.3 | 31.0 | < 0.0001 |
| 3-Multiparous women with a single cephalic pregnancy at ≥ 37 weeks gestation, non-instrumental delivery | 8.2 | 6.9 | 7.0 | 6.7 | 5.6 | 5.0 | < 0.0001 |
| 4-Multiparous women with a single cephalic pregnancy at ≥ 37 weeks gestation, instrumental delivery | 30.7 | 27.1 | 27.6 | 19.3 | 19.4 | 21.7 | 0.0005 |
| 5-All women with a single cephalic pregnancy at < 37 weeks gestation | 15.6 | 15.8 | 15.1 | 9.3 | 10.4 | 8.5 | 0.0001 |
| 6-All women with a single breech pregnancy | 27.2 | 19.8 | 18.9 | 28.3 | 16.8 | 18.5 | 0.12 |
| 7-All women with multiple pregnancy | 17.7 | 15.6 | 20.5 | 11.6 | 6.9 | 8.9 | 0.02 |
| Total | 17.6 | 17.3 | 17.1 | 15.6 | 13.2 | 12.6 | < 0.0001 |
| Level 3 |  |  |  |  |  |  |  |
| 1- Nulliparous women with a single cephalic pregnancy, at ≥ 37 weeks gestation, non-instrumental delivery | 20.0 | 22.3 | 18.0 | 13.7 | 8.0 | 6.4 | < 0.0001 |
| 2- Nulliparous women with a single cephalic pregnancy at ≥ 37 weeks gestation, instrumental delivery | 45.7 | 50.7 | 41.8 | 34.2 | 17.2 | 18.7 | < 0.0001 |
| 3-Multiparous women with a single cephalic pregnancy at ≥ 37 weeks gestation, non-instrumental delivery | 5.1 | 6.9 | 4.0 | 3.3 | 2.5 | 2.6 | < 0.0001 |
| 4-Multiparous women with a single cephalic pregnancy at ≥ 37 weeks gestation, instrumental delivery | 24.2 | 32.9 | 29.3 | 28.6 | 13.2 | 6.8 | < 0.0001 |
| 5-All women with a single cephalic pregnancy at < 37 weeks gestation | 10.7 | 9.2 | 11.6 | 7.3 | 3.4 | 3.6 | 0.003 |
| 6-All women with a single breech pregnancy | 8.5 | 5.4 | 8.2 | 4.6 | 8.2 | 4.2 | 0.54 |
| 7-All women with multiple pregnancy | 47.1 | 25.0 | 23.6 | 25.9 | 12.8 | 7.1 | < 0.0001 |
| Total | 16.1 | 17.6 | 13.6 | 11.5 | 7.0 | 6.8 | < 0.0001 |

*Cochran Armitage Test
