# Supplementary material for: A novel classification for evaluating episiotomy practices: application to the Burgundy perinatal network
Source: BMC Pregnancy Childbirth. 2019 Aug 16;19:300. doi: 10.1186/s12884-019-2424-2 (PMC6698013; doi:10.1186/s12884-019-2424-2)
Supplement: Supplementary file 4 — Table S4. Comparison of episiotomy rates by level of maternity ward according to classification: Burgundy perinatal network data, vaginal deliveries, 2011–2016. (DOCX 19 kb) [file 12884_2019_2424_MOESM4_ESM.docx]

Additional file 4: Table S4: Comparison of episiotomy rates by level of maternity ward according to classification: Burgundy perinatal network data, vaginal deliveries, 2011-2016.

|  | Level of maternities | | | *P** |
| --- | --- | --- | --- | --- |
|  | 1 | 2 | 3 |  |
| 1 – Nulliparous women with a single cephalic pregnancy, at ≥ 37 weeks gestation, non-instrumental delivery | 1,086/4,708 (23.1) | 3,157/13,551 (23.3) | 601/3,980 (15.1) | < 0.0001 |
| 2 – Nulliparous women with a single cephalic pregnancy at ≥ 37 weeks gestation, instrumental delivery | 1,005/1,603 (62.7) | 2,049/5,461 (37.5) | 623/1,920 (32.4) | < 0.0001 |
| 2a – Forceps/ spatula delivery | 784/1,091 (71.9) | 990/1,972 (50.2) | 315/668 (47.2) | < 0.0001 |
| 2b - Vacuum delivery | 221/512 (43.2) | 1,059/3,489 (30.4) | 308/1,252 (24.6) | < 0.0001 |
| 3 – Multiparous women with a single cephalic pregnancy at ≥ 37 weeks gestation, non-instrumental delivery | 582/9,126 (6.4) | 1,727/26,091 (6.6) | 273/6,712 (4.1) | < 0.0001 |
| 4 – Multiparous women with a single cephalic pregnancy at ≥ 37 weeks gestation, instrumental delivery | 196/423 (46.3) | 414/1,725 (24.0) | 107/529 (20.2) | < 0.0001 |
| 4a – Forceps/ spatula delivery | 152/248 (61.3) | 190/464 (40.9) | 48/133 (36.1) | < 0.0001 |
| 4b - Vacuum delivery | 44/175 (25.1) | 224/1,261 (17.8) | 59/396 (14.9) | 0.015 |
| 5 – All women with a single cephalic pregnancy at < 37 weeks gestation | 58/516 (11.2) | 249/1,997 (12.5) | 67/859 (7.8) | 0.001 |
| 6 – All women with a single breech pregnancy | 28/139 (20.1) | 178/834 (21.3) | 19/295 (6.4) | < 0.0001 |
| 7 – All women with multiple pregnancy | 19/93 (20.4) | 62/454 (13.7) | 62/272 (22.8) | 0.005 |
| Total | 2,974/16,608 (17.9) | 7,836/50,113 (15.6) | 1,752/14,567 (12.0) | < 0.0001 |

* Fisher’s exact tests.
