# Supplementary material for: A novel classification for evaluating episiotomy practices: application to the Burgundy perinatal network
Source: BMC Pregnancy Childbirth. 2019 Aug 16;19:300. doi: 10.1186/s12884-019-2424-2 (PMC6698013; doi:10.1186/s12884-019-2424-2)
Supplement: Supplementary file 5 — Table S5. Comparison of episiotomy rates according to hospital status: Burgundy perinatal network data, vaginal deliveries, 2011–2016. (DOCX 18 kb) [file 12884_2019_2424_MOESM5_ESM.docx]

Additional file 5: Table S5: Comparison of episiotomy rates according to hospital status: Burgundy perinatal network data, vaginal deliveries, 2011-2016.

|  | Status of hospitals | | | |  |
| --- | --- | --- | --- | --- | --- |
|  | Private | | Public | | *P** |
| 1 – Nulliparous women with a single cephalic pregnancy at ≥ 37 weeks gestation, non-instrumental delivery | 342/2,421 | 14.1 | 4,502/19,818 | 22.7 | < 0.0001 |
| 2 – Nulliparous women with a single cephalic pregnancy at ≥ 37 weeks gestation, instrumental delivery | 770/1,767 | 43.6 | 2,907/7,217 | 40.3 | 0.01 |
| 3 – Multiparous women with a single cephalic pregnancy at ≥ 37 weeks gestation, non-instrumental delivery | 311/5,042 | 6.2 | 2,271/36,887 | 6.2 | 0.97 |
| 4 – Multiparous women with a single cephalic pregnancy at ≥ 37 weeks gestation, instrumental delivery | 175/626 | 28.0 | 542/2,051 | 26.4 | 0.47 |
| 5 – All women with a single cephalic pregnancy at < 37 weeks gestation | 46/333 | 13.8 | 328/3,041 | 10.8 | 0.1 |
| 6 – All women with a single breech pregnancy | 8/45 | 17.8 | 217/1,223 | 17.7 | 1.0 |
| 7 – All women with multiple pregnancy | 4/55 | 7.3 | 139/764 | 18.2 | 0.04 |
| Total | 1,656/10,289 | 16.1 | 10,906/71,001 | 15.4 | 0.06 |

* Fisher’s exact tests.
